# Live-Attenuated Bacterial Vectors: Tools for Vaccine and Therapeutic Agent Delivery

**DOI:** 10.3390/vaccines3040940

**Published:** 2015-11-10

**Authors:** Ivan Y. C. Lin, Thi Thu Hao Van, Peter M. Smooker

**Affiliations:** School of Applied Sciences, RMIT University, Plenty Road, Bundoora VIC-3083, Australia; E-Mails: tertlelin@gmail.com (I.Y.C.L.); thithuhao.van@rmit.edu.au (T.T.H.V.)

**Keywords:** vaccine, attenuated vector, infectious disease, *Salmonella*, cancer therapy

## Abstract

Genetically attenuated microorganisms, including pathogenic and commensal bacteria, can be engineered to carry and deliver heterologous antigens to elicit host immunity against both the vector as well as the pathogen from which the donor gene is derived. These live attenuated bacterial vectors have been given much attention due to their capacity to induce a broad range of immune responses including localized mucosal, as well as systemic humoral and/or cell-mediated immunity. In addition, the unique tumor-homing characteristics of these bacterial vectors has also been exploited for alternative anti-tumor vaccines and therapies. In such approach, tumor-associated antigen, immunostimulatory molecules, anti-tumor drugs, or nucleotides (DNA or RNA) are delivered. Different potential vectors are appropriate for specific applications, depending on their pathogenic routes. In this review, we survey and summarize the main features of the different types of live bacterial vectors and discussed the clinical applications in the field of vaccinology. In addition, different approaches for using live attenuated bacterial vectors for anti-cancer therapy is discussed, and some promising pre-clinical and clinical studies in this field are outlined.

## 1. Introduction

Vaccination is considered one of the most effective strategies for the prevention of infectious diseases. In the ongoing endeavor to create vaccines against diseases for which none exist, many avenues are being explored. Some of the earliest vaccines were the class termed “attenuated”, so called because although they can infect a host, generally by the normal entry route, their replication is limited and they do not cause disease. They do however induce strong immune responses, and importantly, responses that are appropriate for protection against the pathogen. In the past couple of decades some of these vaccines have been utilized as vectors to deliver heterologous antigens, with a view to vaccinating against both the vector pathogen and the pathogen from which the donor gene is derived. While infectious disease is a terrible human burden, so is cancer. The versatility of live-attenuated bacterial vectors also allows targeted-delivery of therapeutic anti-cancer agents to tumors, which can potentially avoid adverse side-effects present when conventional chemotherapeutic drugs are used. This review will survey live-attenuated bacterial vaccine vectors and examples of their potential for preventing infectious disease, and importantly, for the treatment of cancers.

## 2. Live-Attenuated Bacterial Vaccine Vectors for Infectious Diseases

**Figure 1:**
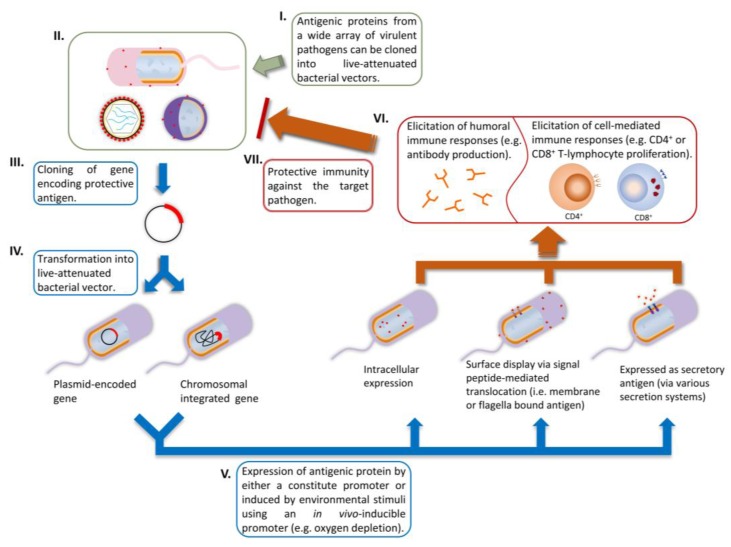
The use of bacterial vectors to vaccinate against pathogens. (**I–IV**) Cloning of heterologous gene and insertion into bacterial vector, either carried on a plasmid or inserted into the chromosome; (**V**) Expression of the heterologous antigen; (**VI**) Elicitation of immune responses; (**VII**) protection against pathogens.

In delivering heterologous antigen, both viral and bacterial vectors can be used, however the subject of this review is the use of bacterial vectors, which is a topic of ongoing research in our laboratory. Bacterial vaccine vectors expressing heterologous antigens and employed as live vaccine vectors have been extensively studied over the last 30 years [1]. Such technology has been used to elicit immune responses against bacterial, viral, protozoan and metazoan pathogens in animal models and clinical studies [1,2]. Bacterial vaccine vectors possess many advantages: (1) easy and inexpensive manufacture with flexible scalability [3]; (2) multiple vaccination routes available, especially the oral mucosal route for elicitation of a broad spectrum immune responses [4]; (3) well-characterized mutations for virulence attenuation [1]; (4) antibiotic-susceptible vaccine vectors are available, thus treatment with an antibiotic is possible if adverse reactions occur [5,6,7]; (5) enteric bacterial vectors have tropism towards lymphoid antigen presenting cells (*i.e.*, dendritic cells and macrophages) in the intestinal mucosal tract, which is an unique asset for developing mucosal vaccines [1,8,9,10,11,12]; (6) they are known to elicit potent adaptive immune responses against homologous and the carried (vectored) heterologous antigens [3,13,14]. Some of the strategies used to employ these vectors for antigen delivery are depicted in Figure 1.

### 2.1. Attenuation of Bacterial Vectors

Historically, attenuation of bacterial vaccine vectors was achieved by chemical mutagenesis or serial passages under laboratory conditions, such as for *Salmonella enterica* serovar Typhi Ty21a [15] and *Mycobacterium bovis* BCG [16]. Modern advances in biotechnology have employed recombinant DNA technology to generate genetically well-defined live-attenuated bacterial vaccine vectors, often achieved by deleting essential genes involved in either virulence regulatory systems (e.g., *phoP* and/or *phoQ* in *Salmonella* spp. [17]) or the aromatic amino acid biosynthesis pathway, also known as auxotrophic mutants (e.g., *aro*A in *Salmonella enteric* serovar Typhimurium [18,19]). Most of the attenuated *Salmonella enterica* vector strains currently used for experimental or clinical studies are auxotrophic strains which are generated via the deletion or mutation of essential genes that are required for the biosynthesis of metabolically essential elements such as aromatic amino acids (*aro*), guanidine (*gua*) or purine (*pur*) [20]. These mutations attenuate the strain whilst preserving its immunogenicity. Such *Salmonella* mutants have shown promising results in animal models. For example, in guinea pigs, oral administration of a *Salmonella* Typhimurium *aro*A mutant expressing chromosomally integrated *Mycobacterium tuberculosis* fusion antigen Ag85B-ESAT6, followed by boosting with a dose of purified Ag85B-ESAT6, successfully reduced the level of *M. tuberculosis* in the lung and spleen to the same extent as the BCG vaccine [21]. Another study demonstrated that a passenger antigen delivered could be detected in Peyers patches and the spleen several days after immunization with attenuated *Salmonella*, even if the expression plasmid was lost soon after administration [22]. Other useful mutations that can render bacteria non-pathogenic are in the genes encoding outer membrane proteins C and F (*omp*C, *omp*F), the cAMP receptor (*cya/crp*) [23,24], as well as mutations in DNA recombination and repair genes (*rec*A, *rec*F) [25].

Among all the vaccine delivery systems that have been developed, including viral particles, attenuated viral vectors, liposomes, ISCOMs and plant-based oral vaccines, live-attenuated bacterial vectors are the most characterized vehicles for mucosal vaccine delivery. Such vaccines can be delivered through oral, intranasal, ocular, rectal, vaginal and pulmonary inhalation routes, and studies have shown elicitation of both mucosal and systemic immune responses [2,26,27,28]. One of the major advantages of using live attenuated vaccine-carriers in regard to mucosal delivery is that they can overcome the obstacles faced by antigen alone at mucosal surfaces. The environment at mucosal surfaces consists of degradative enzymes and extreme pH, which prevents free antigens from reaching target cells. Furthermore, free antigens alone are usually less immunogenic due to poor uptake by mucosal cells [2]. Enteric pathogens are known to survive the mucosal environment by their intrinsic protective mechanisms, and hence are able to protect the heterologous antigen carried with them. Moreover, live vaccine-carriers such as *Salmonella* spp. have been demonstrated to target the intestinal sensing cells (*i.e.*, M cells) that overlay the gut-associated lymphoid tissue (GALT) [29], which is known to play a key role in stimulating mucosal immune responses. In addition, *Salmonella* spp. have the ability to be taken up by phagocytic cells and transverse the reticuloendothelial system, consequently leading to the stimulation of systemic immune responses [30,31,32].

The development of an effective vector depends on the delicate balance between maximal immunogenicity towards the antigens and minimal side-effects [33]. Current studies indicate strains that cause no side-effects are often over-attenuated and thus inadequately immunogenic, even to the homologous (vector) antigens [34,35]. Therefore, careful selection of genes to knock-out for the attenuation of vaccine-carriers is required for the development of robust vaccine systems.

### 2.2. Genetic Stability and Protein Expression of Heterologous Genes

In general, the heterologous gene encoding the vaccine antigen can be either integrated into the bacterial chromosome or expressed from a plasmid. Chromosomal integration can be achieved by locus deletion and replacing with a cassette encoding the heterologous antigen gene; this allows maximum genetic stability, as chromosomal DNA rarely undergoes mutation or deletion [36]. However, chromosomal integration usually results in a single copy of heterologous antigen per bacterium, and it is a challenge to ensure that sufficient antigen is expressed to confer protective immunity [37]. Plasmid-based expression is another option for carrying heterologous antigen. A wide array of plasmid-based expression systems is available for such applications. However, plasmid-based expression systems have two major challenges that have to be overcome to achieve both the required immunogenicity and genetic stability. The metabolic burden associated with plasmid replication can lead to over-attenuation of the vaccine-carrier thus reducing immunogenicity, and spontaneous loss of plasmid is frequent resulting in plasmid-less bacteria rapidly outgrowing plasmid-bearing bacteria and becoming the dominant population in tissues [38]. Various mechanisms have been proposed to enhance plasmid retention: (1) self-transferring plasmid for mobilization of plasmid between bacteria; (2) selective advantage to enhance plasmid inheritance [39]; (3) self-regulating origin for plasmid replication [40]; (4) promote plasmid distribution by an active partitioning mechanism [39]; (5) post-propagation killing of plasmid-less bacteria to ensure a population of only plasmid-bearing bacteria [41]. However, it should be remembered that self-transferring plasmids and the use of clinically relevant antibiotics as selective pressure are strongly discouraged by regulatory authorities due to potential safety concerns [42].

One of the most successful strategies for the prevention of plasmid loss is known as the conditional lethal system. This system maintains the plasmid-bearing bacterial population by encoding replication-essential proteins in the plasmid, so that plasmid-less daughter cells are unable to survive [43]. In one such approach, the *asd* gene that encodes aspartate β-semialdehyde dehydrogenase was used. It is an important enzyme for not only amino acid synthesis, but also for cell wall synthesis [43]. Consequently, in an *asd-*deleted bacterial host, harbouring an *asd*-encoding plasmid is essential for survival. This particular system has been successfully demonstrated in a live attenuated *S*. Typhimurium to deliver a variety of heterologous antigens including a viral peptide from HBV [44], and F1-Ag and V-Ag antigens derived from *Yersinia pestis* [45]. The *S.* Typhimurium vaccine induced potent humoral immune responses, including serum IgG and secretory IgA in mucosally immunized mice.

### 2.3. Controlling Antigen Expression and Antigen Compartmentalization

Another important aspect of using a vaccine carrier to deliver heterologous antigen is how the protein expression is controlled and compartmentalized. Regulating the level and location of heterologous antigen expression can have a significant impact on the immunogenicity of the vaccine. Incorporating an appropriate expression promoter is the key to regulating the desired level and timing of antigen delivery, and to confer optimal immune responses [46]. Early studies conducted by Hohmann *et al.*, 1995 [46] demonstrated that an antigenic protein expressed from a constitutive promoter encoded in the *S*. Typhimurium chromosome was incapable of inducing a protective antigen-specific immune response. In contrast, the use of *in vivo*-inducible promoters such as P*_pagC_*, which only induces antigen expression after the bacterial cell has been phagocytosed by macrophages, resulted in the induction of a strong serum IgG response against the same antigen [46]. It was however suggested that although the constitutive promoter confers high levels of heterologous antigen expression, there is an overall increase in metabolic burden to the vaccine carrier, which compromises immunogenicity [47].

Several other *in vivo*-inducible promoters which respond to environmental stimuli *in vivo* have been exploited for the purpose of controlling protein expression in live-attenuated bacterial vaccine vectors, and include those induced by oxidizing agent availability [48], low iron concentration [49] and low magnesium concentration [50]. Chu *et al.*, 2015 [51] reported the use of quorum sensing genes from *Vibrio fischeri* with iron uptake regulons, and a synthetic binary regulation system was designed for *Edwardsiella tarda*. The vaccine construct carried a protective antigen glyceraldehyde-3-phosphate dehydrogenase (GAPDH) from the fish pathogen *Aeromonas hydrophila* LSA34. A challenge study was carried out in Turbot (*Scophtalmus maximus*), and most vaccinated fish survived the challenge. This is an example where a well-controlled *in vivo*-inducible promoter can significantly enhance vaccine immunogenicity.

Correct protein folding and localization is essential for inducing protective humoral responses against conformational epitopes. Several strategies have been developed, which include the export of the antigen to the extracellular space and direct surface display [52,53]. Kang and Curtiss, 2003 [54] demonstrated that in orally immunized mice, the antigen-specific humoral response of an attenuated *S.* Typhymurium carrying PspA derived from the *S. pneumonia* surface protein was enhanced 10,000-fold by incorporating the secretion signal from β-lactamase compared with the unfused PspA construct. Surface display is an attractive means to present heterologous antigen to the host immune system because of the potential to elicit potent humoral immunity. Heterologous genes can be fused into the gene encoding bacterial outer membrane proteins such as OmpA [55], LamB [56] or flagellin [57]. The fusion protein that contains the heterologous antigen is then presented on the bacterial outer membrane for immunological stimulation. Another approach for surface display is to utilize the bacterial autotransporter (AT) system. The ATs are abundant proteins encoding many virulence factors of Gram-negative bacteria, and are responsible for exporting the N-terminal domain of the fusion protein to the bacterial outer membrane [57].

### 2.4. Current Achievements and Perspectives

Pathogenic microorganisms are of particular interest for developing live-attenuated bacterial vectors, as most of them are well adapted to the environment of the mucosal surface, and initiating the infection process. Therefore, the early live bacterial vectors were constructed from pathogenic microorganisms such as *Salmonella*, *Listeria* [58,59], and *Mycobacterium* [60]. However, these attenuated pathogenic strains retain a level of residual virulence (essential for the induction of immune responses) that render them unsuitable for the vaccination of individuals such as infants, the elderly or immunocompromised patients [60]. In an effort to allay these concerns, non-pathogenic microorganisms, such as lactic acid bacteria, have been exploited as antigen delivery vehicles. The characteristics of the main microorganisms used for developing live-attenuated bacterial vector vaccines are summarized in Table 1, and some examples of live-attenuated bacterial vector vaccines that have reached preclinical evaluation and Phase I clinical trials are listed in Table 2. These studies clearly point to the promise of these approaches, with strong and protective humoral and cellular immunity generated by these vaccines.

In addition to these examples, there are several further innovations that have been developed to enhance immunogenicity and the safety profile of live-attenuated bacterial vaccine vectors.

The co-delivery of cytokines and heterologous antigen can enhance vaccine immunogenicity [61]. Cytokines are crucial molecules that orchestrate innate and adaptive immunity, as well as the development of immunological memory [62]. Byrd *et al.*, 2002 [61] constructed a recombinant *Streptococcus gordonni* strain that expressed either murine interleukin-2 (IL-2) or interferon-γ (IFN-γ) in addition to a surface anchored model protein (the conserved C-repeat region of the M6 protein derived from *Streptococcus pyogenes*). In their experiment, in mice that were subcutaneously immunized with the *S. gordonii* strain, cytokine co-expression modulated the systemic immune response. Furthermore, *in vivo* expression of cytokines delivered using live-attenuated bacterial vectors has been successfully employed for therapeutic purposes in many studies. One such example was demonstrated by Braat *et al.*, 2006 [63], in which genetically modified *Lactococcus lactis* encoding mature human interleukin-10 (IL-10) was used for the treatment of Crohn’s disease. A Phase I trial demonstrated that the vector was not only safe for humans with minor adverse effects, but a decreased disease severity was also observed. Cytokine delivery using live-attenuated bacterial vectors has also been applied for tumor prevention and/or therapeutic measures; these will be discussed later in the review.

Significant progress in the development of live-attenuated bacterial vectors as prophylactic vaccines has been achieved over the past few decades. Many studies have demonstrated how effective and powerful these vectors can be when used to promote human and animal health. The research field of live-attenuated bacterial vectors is still relatively young, and there are new vectors and expression systems under development. Therefore, it is not difficult to imagine that some of these live-attenuated bacterial vectors will become available for either the prevention or treatment for diseases in the near future.

**Table 1:** Characteristics of the main microorganisms used for the development of live-attenuated bacterial vector vaccines.

Vector	Target Host Cell	Advantages	Limitations/Concerns	Ref.
*Enteric pathogens*				
*Listeria monocytogenes*	Intestinal epithelial cells and non-phagocytic cells such as hepatocytes are primary invasion sites before systemic dissemination. Intracellular pathogen.	Ability to present homologous and heterologous antigens to both endogenous and exogenous antigen-presenting pathways, thus eliciting CD4^+^ and CD8^+^ T-lymphocyte responses.	Wild-type *Listeria* can cause serious and potentially lethal disease, especially in immunocompromised individuals. Severe attenuation to ensure safety could over-attenuate and lead to poor immunogenicity. Possible reversion to pathogenic state.	[64]
*Salmonella* spp.	M cells as primary invasion site, and taken up by phagocytic cells for systemic dissemination. Intracellular pathogen.	Among the first bacteria used as vaccine-carrier to deliver heterologous antigens, well-established protocol for genetic manipulation. Stimulate humoral immune responses and induce serum IgG and secretory IgA antibody. Elicits both cytotoxic and memory T-lymphocyte responses.	Pre-existing immunity could decrease immunogenicity. Possible reversion to pathogenic wild-type.	[14,29,60,65,66,67]
*Vibrio cholerae*	M cells and intestinal epithelial cells. Extracellular pathogen.	Ability to adhere to M cells and other epithelial cells without further invasion (decreased pathogenicity). Ideal for delivering antigens from luminal pathogens rather than systemic infections.	Unable to elicit systemic and potent cell-mediated immune responses. Possible reversion to pathogenic state.	[68]
*Commensal organisms*				
*Lactobacillus* spp.	Colonize gastrointestinal and uro-genital mucosa. Normal flora.	Non-pathogenic bacteria. Ability to stimulate antigen-specific immune responses via intranasal and oral routes. Special interest for the development of sexually transmitted diseases vaccines.	Unable to elicit cell-mediated immune responses.	[69,70,71]
*Staphylococcus* spp.	Colonize oral, nasal and uro-genital mucosa. Normal flora.	Food grade bacteria with intrinsic safety profiles. Stable colonization by a single intranasal or oral inoculation for more than two months. Ability to stimulate systemic immune responses against heterologous antigens. Strains such as *S. carnosus* have low extracellular proteolytic activity, which facilitates stable display of heterologous antigens.	Possible cause of pyelonephritis and endocarditis. Pre-existing immunity could decrease immunogenicity.	[72,73,74]

**Table 2:** Examples of live-attenuated bacterial vector vaccines that have reached preclinical evaluation and Phase I clinical trials.

Vector	Mutation/Attenuation	Heterologous Antigen	Inoculation Route	Target Host	Outcome	Ref.
*Listeria monocytogenes* BMB72	Δ*act*A Δ*act*B	Influenza A nucleoprotein	Oral and transcutaneous	Human	All volunteers who received the vector vaccine developed detectible mucosal immune responses to listerial antigens, but not to the heterologous influenza antigen.	[75,76]
*Bordetella pertussis* BPZE1	Lacking *dnt* gene and producing inactive pertussis toxin and reduced tracheal cytotoxin.	SP70 derived from enterovirus 71	Intranasal	Mouse	Strong and sustained systemic anti-SP70 antibody response was observed in nasally immunized mice.	[77]
*Listeria monocytogenes* XFL-7	*prf*A-defective	HPV-16 E7 antigen	Intravenous	Human	HPV-16 E7-specific T lymphocyte responses were elicited	[78]
*Salmonella* Typhi	Δ*ssa*V Δ*aro*C	*Escherichia coli* heat labile toxin (LT-B)	Oral	Human	Humoral immune responses to LT-B and *S.* Typhi lipopolysaccharide were observed in 67 and 97% of subjects.	[79]
*Salmonella* Typhi Ty21a	ΔgalE with undefined attenuating mutations	OprF-Oprl derived from *Pseudomonas aeruginosa*	Oral and intranasal	Human	A significant elevated IgA and IgG antibody levels in the lower airways was observed.	[80]
*Salmonella* Typhi Ty21a	Δ*gal*E with undefined attenuating mutations	Urease or HP0231 derived from *Helicobactor pylori*	Oral	Human	T cell-mediated immunity against *H. pylori* was elicited in immunized subjects.	[81]
*Salmonella* Typhi Ty21a	Δ*gal*E with undefined attenuating mutations	O-Ps derived from *shigella dysenteriae*	Oral	Human	Protective immunity was elicited against challenge assay with *S. dysenteriae*	[82]
*Vibrio cholera*	ΔCTA	Cholera toxin-B	Oral and intranasal	Mouse and rabbit	Cholera toxin has >80% identity to *E. coli* (ETEC) heat-labile protein. Neutralizing antibody responses against ETEC heat-labile toxicity was observed in vaccinated mice and rabbits.	[83]

## 3. Live-Attenuated Bacterial Vectors for Cancer Treatment

There are ongoing endeavors to search for cancer therapies that exhibit greater treatment efficacy, specificity, and selectivity. *In vivo* therapeutic cancer vectors that directly deliver heterologous antigen or genes encoding various anti-cancer molecules have been studied and developed for such purposes. Some microorganisms have been shown to selectively replicate within solid tumors or preferentially colonize the tumor micro-environment, hence providing an attractive platform for tumor targeting therapy. This section will summarize the use of various bacterial vectors in development as a novel cancer treatment strategy.

### 3.1. Tumor-Targeting Ability of Bacterial Vectors

**Figure 2:**
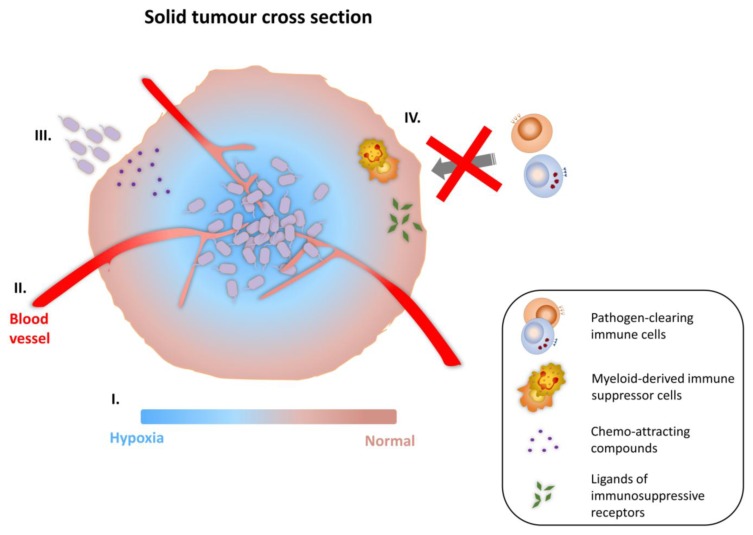
Tumor-targeting ability of bacterial vectors. (**I**) The hypoxic microenvironment in necrotic areas of solid tumor favours the colonization and proliferation of facultative anaerobes and obligate anaerobes; (**II**) Increased entrapment in the organisationally compromised and leaky vasculature, caused by neo-angiogenesis; (**III**) Chemo-attracting compounds present in necrotic areas (e.g., aspartate, serine, citrate, ribose or galactose) allow bacterial vectors to taxi toward tumors; (**IV**) Compromised pathogen clearance due to the presence of myeloid-derived immune suppressor cells and the accumulation of ligands of immunosuppressive receptors in the tumor micro-environment.

There are a number of bacterial species that have been demonstrated to target solid tumors, and lead to therapeutic effects in tumor-bearing rodent models. Generally, anaerobic bacteria are employed. Amongst the bacterial species used, *Clostridum* and *Bifidobacterium* are strict anaerobes, whereas *Salmonella* and *Listeria* are facultative anaerobes. It was initially believed that the hypoxic micro-environment present in necrotic areas of solid tumors was the primary driver of their tumor-targeting ability. However, recent investigations have uncovered various other micro-environment characteristics which might favor the preferential proliferation and colonization of bacteria in tumors [84]. These are illustrated in Figure 2. Kasinskas and Forbes, 2007 [85] suggested that the chemo-attracting compounds present in necrotic regions (e.g., aspartate, serine, citrate, ribose or galactose) produced by quiescent cancer cells are important contributing factors for bacterial chemotaxis towards tumors. Furthermore, other unique micro-environment properties found within solid tumors such as aberrant neo-vasculature and localized immunosuppression are also believed to be factors involved in bacterial tumor-targeting. Actively growing tumors promote the formation of new blood vessels, a process known as neo-angiogenesis. These newly formed blood vessels exhibit malformed vascular structure, being highly disorganized with impaired endothelial linings and blind ends. As the result, these blood vessels are “leaky”, and may allow circulating bacteria to escape from tumor vasculature and lodge locally in tumor tissue [86,87]. In addition, multiple tumor-induced immunosuppressive mechanisms have been proposed to explain the preferential colonization and proliferation of bacterial vectors in tumors. Tumor masses may contain myeloid-derived immune suppressor cells, which alter and reprogram the activation of macrophages and antigen presenting cells (APCs) [88]. Furthermore, there are studies demonstrating direct inhibition of immune cell-activating transduction via the accumulation of ligands of immunosuppressive receptors in the tumor micro-environment [89,90]. Each of these structural and immune dysregulation events in tumors creates an environment for bacteria to flourish.

### 3.2. Strategies for Cancer Therapy

Various strategies been developed for bacteria-mediated anti-tumor therapy, and some of these have entered pre-clinical and/or clinical phase trials for evaluation. In the past, microorganisms such as *Salmonella* [91] and *Clostridium* [92] were noted for their intrinsic oncolytic capacity, and can induce tumor growth retardation. However, despite the initial tumor retardation and necrosis that was observed after application of these microorganisms, the tumor can often re-grow after treatment. Therefore, solely relying on the intrinsic anti-tumor ability of these oncolytic vectors is not sufficient; consequently many studies have addressed methods to improve the therapeutic effects. Several genes have been exploited and genetically engineered into these vectors. According to their mode of action, these genes can be categorized as acting via direct killing or immunomodulation (Table 3).

**Table 3:** Examples of therapeutic genes carried by bacterial vectors for anti-cancer treatment.

Mode of Action	Therapeutic Approach	Example of Passenger Gene (or Antigen)	Delivery Vector	References
Direct cell killing or tumor growth retardation	Intrinsic oncolytic vector	None	Clostridium spp. Salmonella spp.	[91,92,93,94,95,96]
	Anti-angiogenic molecule	Vascular endothelial growth factor receptor 2 molecule (fetal liver kinase-1)	*L. monocytogenes* *S.* Typhimurium	[97,98]
		Endostatin	*B. longum* *B. adolescentis*	[99,100,101,102]
			*S. choleraesuis*	[103]
	RNA interference	Anti-bcl2 shRNA	*S.* Typhimurium	[104]
		Anti-MDR1 siRNA	S. Typhi	[105]
	Cell death inducer	Fas ligand	*S.* Typhimurium	[106]
		HylE cytolynsin	*S.* Typhimurium	[107]
		TNF-related factor apoptosis ligand (TRAIL)	*S.* Typhimurium *B. longum*	[101,108,109]
		Apoptin	*S.* Typhimurium	[110]
	Pro-drug activating enzyme (+drug)	Herpes Simplex Virus thymidine kinase (HSVtk) + ganciclovir	*S.* Typhimurium	[111,112]
			*B. infantis*	[113]

**Figure 3:**
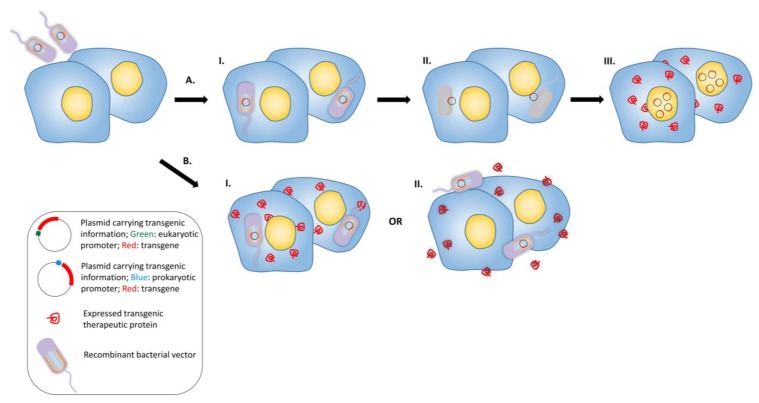
Bactofection into tumors. (**A**) Bacteria are used as a vector to deliver the genetic information into the eukaryotic cell. Bacterial vectors that possess plasmid (each bacterial vector can carry multiple copies of transgenic plasmid) carrying a transgene are administered into the target tissue, I.: The vectors penetrate into the cells. II: Vectors undergo lysis and the plasmids are released into the cytoplasm, III: The released plasmids enter the nuclei and the therapeutic transgene is expressed by eukaryotic transcription and translation mechanisms; (**B**) Alternative gene therapy: recombinant bacterial vectors express the recombinant therapeutic protein *in situ* intracellularly or in the intercellular space. Recombinant bacterial vector that possess plasmid carrying the transgene are administered into the target tissue and either enter the cells or stay in the intercellular space; I: The transgene is expressed and secreted after entering the cell, or; II: Bacteria do not enter the eukaryotic cell, but express the therapeutic transgene in the intercellular space.

Bactofection is the term to describe the technique of using bacteria as a vehicle to deliver therapeutic cargo into the target organism, cell, organ or tissue [114]. This can be by the delivery of a plasmid to be transferred to the host cell for production of heterologous antigen, or production of the heterologous antigen (often followed by secretion) by the bacterial vector itself. Figure 3 represents the process for the delivery of a foreign gene. Several invasive bacterial species have been demonstrated to successfully transfer a eukaryotic expression plasmid into mammalian cells, such as *S*. Typhimurium [104,115,116,117], *Shigella* [118], *L. monocytogenes* [119] and recombinant *E. coli* [120]. Amongst them, *S.* Typhimurium is the most extensively studied vector for bactofection, demonstrating promising therapeutic expression and anti-tumor effects [104,106,108,121]. To date, the mechanisms involving DNA transfer by bacterial vectors are still not fully understood for many species, and proposed mechanisms are deduced from the inherent bacterial invasion properties (*i.e.*, pathogenic pathway) and the cell type involved [122]. Basically, invasive microorganisms such as *S*. Typhimurium and *L. monocytogenes* are favored as bactofection vectors, but non-invasive microorganisms such as *Bifidobacterium longum* have also received attention due to their outstanding safety profiles [99,101,123,124,125]. For bactofection, two major engineered species are being widely exploited in pre-clinical and clinical studies: pathogens with intrinsic intracellular tropism, *S*. Typhimurium and *L. monocytogenes*. Although both attenuated vectors have shown promising potential as a gene delivery vehicles, one major difference between them has determined their fate in the development of anti-tumor bacterial vectors.

In these gene delivery methods, DNA is transferred to mammalian cells and subsequently transcribed by host cell-machinery within the nucleus. Although these DNA molecules can be delivered straight into the cell nucleus by employing viral vectors [126], plasmid-based DNA transfer systems often result in limited effectiveness, as the spontaneous trafficking of plasmid DNA into the cell nucleus is rate-limiting and inefficient [127]. However, RNA can also be delivered by bacteria. Schoen *et al.*, 2005 [128] demonstrated the use of self-destructing *L. monocytogenes* to release ready-to-translate enhanced green fluorescent protein (EGFP)-encoding mRNA into the cytosol of epithelial cells. Their investigation revealed an enhanced EGFP expression level over the conventional plasmid DNA delivery strategy.

In terms of production of the protein by the vector itself, *L. monocytogenes* has a major advantage. It is equipped with two unique virulence factors known as listeriolysin O and phospholipase C, which enables the bacteria to escape phagosomal degradation. Furthermore, an actin polymerase known as ActA allows *L. monocytogenes* to be internalized by adjacent cells, hence the intracellular lifecycle enables the antigens secreted by *L. monocytogenes* to be available for both MHC class I and class II presentation on the cell surface [129,130]. In contrast, antigen secreted by *S.* Typhimurium is processed as exogenous antigen and presented mainly by MHC class II molecules. This is because *S.* Typhimurium lacks mechanisms to escape the phagosome, hence their intracellular survival and replication is restricted to the organelle, and secreted antigens are seen as exogenous. Finally, as demonstrated by Darji *et al.*, 1997 [22,131], even though the expression plasmid carried by *S.* Typhimurium is lost soon after oral administration, the encoded passenger antigen can still be detected.

Different therapeutic strategies were therefore developed according to their unique characteristics. As summarized in Table 3, *L. monocytogenes* has been receiving particular interest in delivering defined tumor antigens. This is because of their capacity to deliver heterologous antigen through the endogenous pathway, consequently presenting antigenic peptides through the MHC class I pathway, leading to the activation of antigen-specific CD8+ CTLs and breaking immune tolerance [132,133]. In 2009, Stark *et al.*, [134] evaluated the differential tumor protection between these two bacterial vectors by constructing *L. monocytogenes* and *S*. Typhimurium, both expressing ovalbumin (OVA). Although both vectors successfully induced functional OVA-specific CD8+ T-lymphocyte responses that expressed IFN-γ *in vivo*, only OVA-expressing *L. monocytogenes* immunized mice were protected against B16-OVA melanoma tumors.

### 3.3. Anti-Angiogenesis

The growth of solid tumors is known to be angiogenesis-dependent and this is often observed in hypoxic and necrotic areas. Therefore, in the quest for more efficient anti-tumor drugs, blocking tumor angiogenesis would be a promising intervention point [135]. Jia *et al.*, 2005 [136] successfully demonstrated this by administering attenuated *S*. Typhimurium VNP20009 in combination with endostatin (an endogenous angiogenesis inhibitor) for the treatment of malignant melanoma in a murine model. Therapy using bacteria or endostatin alone had little or no effect on tumor growth. However, the combined therapy was very effective at retarding tumor growth, and was associated with marked tumor necrosis. Their investigation suggested that tumor-targeting bacteria can administer drugs to poorly perfused tumor areas. Lee *et al.*, 2004 [103] constructed *Salmonella choleraesuis* carrying endostatin in a eukaryotic expression vector, and the vector was intraperitoneally administered into tumor-bearing mice. The construct significantly inhibited tumor growth by 40% to 70%, and markedly prolonged survival was observed [117]. In addition, immunohistochemical studies indicated reduced intratumoral microvessel density, and enhanced infiltration of CD8+ T-lymphocytes. *B. longum* and *Bifidobacterium adolescentis* vectors engineered to delivery plasmid-encoded endostatin showed similar anti-tumor results in tumor-bearing mice [99,100], and some promising preclinical results have been demonstrated in various tumor models [100,102,137,138]. Finally, another strategy used to inhibit angiogenesis is to break tumor immune tolerance by stimulating the host immune system to recognize tumor antigens such as endothelial growth factor receptor 2. Seavet *et al.*, 2009 [98] constructed *L. monocytogenes* to carry polypeptides from mouse vascular endothelial growth factor receptor 2 molecule which was fused with a microbial adjuvant, listeriolysin O. In immunized mice, the construct was able to elicit strong anti-tumor CTL responses, was able to eliminate some established breast tumors, and reduced microvessel density in the remaining tumors. Attenuated *S*. Typhimurium strains SL3261 and VPN20009 have also been used to mediate anti-angiogenesis therapy with promising results [97,136]. Taken together, these studies point towards a promising future in the use of bacterial vectors to limit angiogenesis in tumors.

### 3.4. RNA Interference

**Figure 4:**
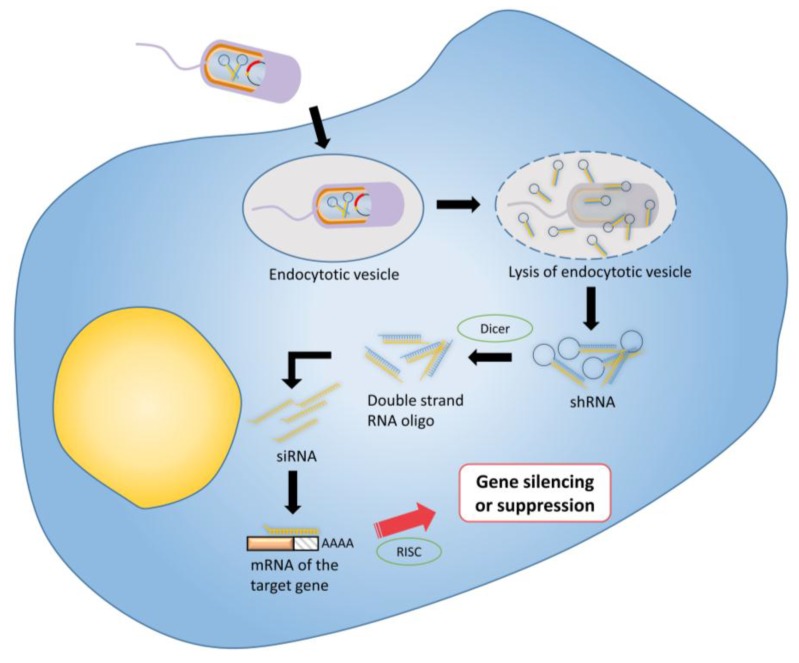
Tumor therapy using RNA interference: Bacterial vectors are transformed with an shRNA-encoding vector for intra-bacterial transcription. shRNA are expressed inside the vectors before release into the target tumour cell’s cytoplasm. Following bacterial lysis, the shRNA molecules are cleaved by Dicer into the corresponding siRNA molecules. The anti-sense strand of the siRNA specifically binds with its target mRNA, which is then degraded by the RNA-induced silencing (RISC) complex resulting to a post-transcriptional gene silencing or suppression.

RNA interference (RNAi) is a highly efficient regulatory process that mediates post-translational gene silencing in most eukaryotic cells; it silences the expression of targeted genes via distinct messenger RNA degradation pathways [139]. RNAi represents a promising new approach to mediate and manage a variety of diseases and medical conditions, including viral infection, cancer and immune disorders [140,141]. Three different forms of RNA molecules are involved in mediating gene regulation, which are microRNA (miRNA), short hairpin RNA (shRNA), and small interfering RNA (siRNA) [140]. However, difficulties in the *in vivo* delivery of RNAi for therapeutic purposes are major limitations in this rapidly expanding field. This is because small interfering RNA molecules are negatively charged polymers that cannot efficiently enter cells, and undergo rapid degradation in the extracellular space [142,143]. A number of bacterial vectors have been exploited and shown to be an efficient, cost-effective and safe strategy for delivering RNAi to malignant tumors. Figure 4 shows the general approach. Zhao *et al.*, 2005 [144] and Xiang *et al.*, 2006 [145] independently demonstrated the concept by showing that siRNA molecules expressed by an invading *E. coli* strain could elicit an RNAi effect in mammalian cell cultures. Furthermore, Xiang and colleague successfully induced significant gene silencing effects in mouse intestinal epithelium and human colon xenografts when administered with *E. coli* engineered to carry shRNA against CTNNB1 (catenin beta-1). Overexpression of CTNNB1 is often associated with cancers including hepatocellular carcinoma, colorectal carcinoma and endometrial cancer [146]. Xiang’s pioneering study provided the first evidence of transfer of RNAi effector molecules from bacterial vectors to mammals *in vivo*, and this delivery strategy is termed transkingdom RNAi. Following these studies, Jiang *et al.*, 2007 [105] constructed attenuated *S.* Typhi as an *in vivo* delivery vector for multidrug-resistance gene (MDR1) siRNA, and successfully transferred MDR1 siRNA to tumors. Zhang *et al.*, 2007 [147] evaluated the combined effects of an oncolytic vector together with an RNAi molecule by systemic administration of *S.* Typhimurium against tumor transcription factor STAT3. Their study revealed greater therapeutic efficiency in combined therapy compared with treatment using the vector alone.

Bacterial vectors have also been exploited to deliver short-hairpin RNA for therapeutic gene-silencing effects. Yang *et al.*, 2008 [104] successfully delayed tumor growth and prolonged survival in a murine melanoma model by orally administering *S.* Typhimurium carrying a short-hairpin RNA against the anti-apoptotic protein bcl-2. Blache *et al.*, 2012 [148] developed *S.* Typhimurium transformed with an shRNA plasmid against indoleamine 2,3-dioxygenase 1 (IDO), an immunosuppressive molecule present in the tumor-associated microenvironment. Upon systemic administration in tumor-bearing mice, anti-IDO shRNA silenced host IDO expression and resulted in considerable intratumoral cell death with significant tumor infiltration by polymorphonuclear neutrophils. Blache and colleagues also presented an interesting finding whereby silencing of host IDO expression significantly enhances *S.* Typhimurium colonization, suggesting intratumoral expression of IDO mediates the immune response to *S.* Typhimurium [148]. In summary, many promising RNAi-mediated anti-tumor therapeutic strategies have been developed, and further investigation will expand potential applications.

### 3.5. Cell Death Inducer

**Figure 5:**
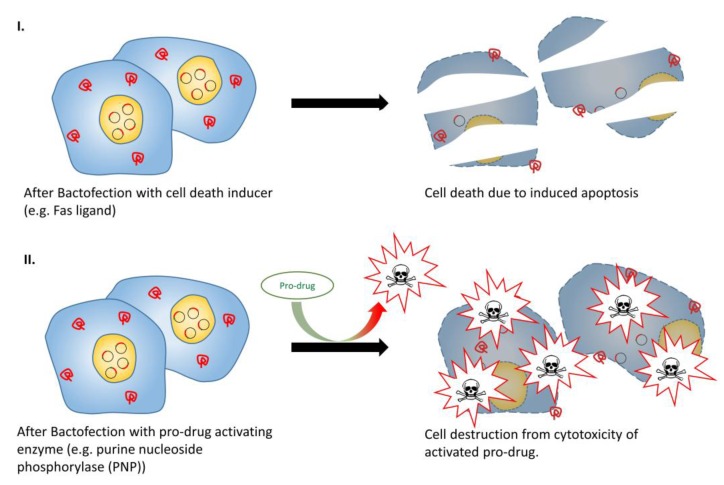
Tumor therapy with cell death inducer or pro-drug activating enzymes. (**I**) Cancer cells transfected with apoptosis inducers such as Fas ligand could lead to apoptosis in Fas-sensitive cells; (**II**) Cells transfected with pro-drug activating enzyme allow more specific and localised cell destruction, even upon systemic pro-drug administration.

In the context of directly inducing cell death, the most straightforward approach features a bacterial vector to deliver and transfect a suitable cytotoxic gene to tumor cells (Figure 5, part I). Fas ligand (FasL) is a membrane protein that belongs to the family of tumor necrosis factor proteins, which can initiate an apoptotic signal in Fas-sensitive cells [149]. Attenuated *S.* Typhimurium has been used to express FasL, with intravenous administration of this vector resulting in tumor growth inhibition in murine breast carcinoma and colon carcinoma models [106]. TNF-related apoptosis-inducing ligand (TRIAL) is another potent apoptotic agent. Chen *et al.*, 2012 [109] and Ganai *et al.*, 2009 [108] conducted separate studies by incorporating TRAIL-expressing plasmids in *S.* Typhimurium under the control of the prokaryotic hypoxic-inducible NirB promoter and radiation-inducible RecA promoter, respectively. Both vectors were able to retard tumor growth and prolonged survival in a murine tumor model. In addition, co-administration of *B. longum* expressing IL-2 and *B. longum* expressing TRAIL in tumor-bearing mice was evaluated, and significantly enhanced anti-tumor effect and prolonged survival was observed when compared with administration with either constructs alone [101]. Ryan *et al.*, 2009 [107] demonstrated an alternative approach to induce tumor cell death, which employed attenuated *S.* Typhimurium carrying a novel cytotoxic protein (HlyE) under the control of FF + 20*, a highly hypoxic-inducible promoter derived from the semi-synthetic bacterial promoter, FF + 20. This resulted in a considerable increase in tumor necrosis and tumor growth retardation in murine mammary tumors. Lastly, more recent investigations have looked into the application of the chicken anemia virus derived protein “apoptin” as a passenger protein for vector-mediated anti-cancer treatment [150]. Guan *et al.*, 2013 [110] successfully prolonged host survival in a syngeneic nude murine tumor model with marked tumor growth retardation and reduced microvessel density after systemic administration of apoptin-expressing *S.* Typhimurium.

### 3.6. Pro-Drug Activating Enzyme + (Drug)

Bacteria-mediated anti-tumor therapies using indirect cytotoxic genes have also shown promise. Gene-directed pro-drug therapy (GDPT) is a two-step approach to deliver anti-cancer effects (Figure 5, Part II). In the first step, a drug-activating enzyme is delivered and expressed in the tumor, whilst in the second step, a non-toxic pro-drug is administered which is selectively activated by the expressed drug-activating enzyme. The net benefit is that a systematically administered pro-drug can be converted locally to a cytotoxic drug, and deliver highly localized anti-tumor effect [151,152]. Several enzyme/prodrug systems are available for such anti-tumor approaches. An early attempt at GDPT was the combination of Herpes Simplex Virus thymidine kinase (HSVtk) and an antiviral drug ganciclovir (GCV). HSVtk phosphorylates GCV by a cellular kinase to produce GCV triphosphate, which interrupts DNA synthesis during S phase, resulting in cell death [154]. Pawelek *et al.*, 1997 [112] successfully demonstrated GCV-mediated, dose-dependent suppression of tumor growth and prolonged survival in mice bearing B16F10 melanoma upon administering HSVtk-expressing attenuated hyper-invasive *S.* Typhimurium. A similar study using *Bifidobacterium infantis* as the HSVtk-expression vector showed similar anti-tumor activity in a rat model of bladder cancer [113]. Moreover, a co-expression, bi-gene GDPT system was investigated by Zeng *et al.*, 2012 [111]. Melanoma-bearing mice were administered with *S.* Typhimurium co-expressing *Mycobacterium tuberculosis* heat shock protein 70 (mtHS70) and HSVtk, and upon administering GCV, increased IFN-γ levels within tumor tissue and marked tumor growth retardation was observed. Some other commonly studied enzyme/pro-drug combinations include *E. coli* cytosine deaminase (eCD) in conjunction with 5-fluorocytosine [96,154,155,156,157], nitroreductase (NTR) in conjunction with the CB1954 prodrug [158,159], and purine nucleoside phosphorylase (PNP) in conjunction with 6-methylpurine 2’-deoxyriboside (MePdR) [116,160]. Of these, the use of eCD in conjunction with 5-fluorocytosine yielded the most promising results. A small pilot trial used attenuated *S.* Typhimurium expressing eCD for patients with refractory cancer, and demonstrated not only the safety profile of the vector, but also the conversion of 5-fluorocytosine to cytotoxic 5-fluorouracil, resulting in noticeable anti-tumor effects in two- thirds of the subjects [161]. Although GDPT is still in its infancy, it is foreseeable that with continuous technological improvements, innovative designs of both vector and enzyme/pro-drug systems will emerge in the near future and it will evolve into a successful routine therapeutic regime for cancer.

### 3.7. Immune Stimulatory Molecules

In-depth understanding of tumor immunology facilitates the design of vector-mediated immune-based therapies for cancer. Tumor cells are known to inhibit or down-regulate immune responses by three major mechanisms: attracting immunosuppressive lymphocyte populations, secreting immunosuppressive cytokines and expression of surface molecules which inhibit immune responses by inducing apoptosis in tumor-infiltrating lymphocytes [90,162,163,164,165]. Cancer immunotherapy approaches focus on mediating anti-tumor effects through either direct or indirect intervention of various effector immune cells, which include B-lymphocytes, CD8+ and CD4+ T-lymphocytes and natural killer cells [133,165,166,167]. Live-attenuated bacterial vectors have been exploited to mediate tumor cells or other cells to express pro-inflammatory or inflammatory cytokines that can enhance anti-tumor activity in various lymphocytes [86,136,166,167]. Several *S.* Typhimurium and *B. longum* vectors carrying immunostimulatory molecules have been experimentally tested in a variety of tumor models evaluating the anti-tumor immunotherapeutic effects, resulting in various degrees of tumor retardation or reduction. Some promising results were obtained from vectors carrying immunostimulatory molecules CLL21, granulocyte colony-stimulating factor (GCSF), IL-2, IL-18, TNF-α or LIGHT [101,125,166,167,168,169,170]. Preclinical studies have also used IL-4-expressing *S.* Typhimurium in combination with IL-18-expressing *S.* Typhimurium, resulting in significantly increased serum IFN-γ and strong anti-tumor effects in melanoma-bearing mice [171].

### 3.8. Tumor Antigen

The tendency of tumor-associated antigens to induce immune tolerance rather than eliciting active T-lymphocyte responses is a major obstacle to tumor immunotherapy. The cause of immune tolerance is associated with the initial presentation of these antigens to the immune system by tumor cells in the absence of co-stimulatory molecules [172,173]. The pathogen-associated molecular patterns (PAMPs) possessed by attenuated bacterial vectors have been exploited to break tumor-induced immune tolerance with some positive results [133,174]. The large majority of anti-tumor vaccine studies have utilized attenuated *S.* Typhimurium and *L. monocytogenes* to deliver tumor-associated antigens [175,176]. In the latter case, the intracellular lifecycle of *L. monocytogenes* is attractive in cancer vaccine development; as its cytoplasmic location is advantageous for antigens to be processed directly and presented by MHC Class I, leading to priming antigen-specific CD8+ T-lymphocyte responses, consequently breaking tumor-induced immune tolerance [133,177]. A number of attenuated *L. monocytogenes* have been developed for expressing a wide range of tumor-associated antigens, such as Her-2/neu [178,179], HPV-16 E7 antigen [78,180,181,182], melanoma associated antigen [183] and prostate-specific antigen [184]. In addition, the tumor-associated antigens carried by *L. monocytogenes* are often fused with listerial virulence factors, such as listerolysin O or ActA, which possess motif sequences rich in proline, glutamic acid, serine and threonine residues (PEST domains); these positively charged residues can direct the fused proteins to proteosomes for degradation and presentation of processed CD8+-specific peptides via the MHC Class I [177]. Sewell *et al.*, 2004 [182] conducted a comparative study using *L. monocytogenes* carrying HPV-17 E7 antigen against *L. monocytogenes* carrying HPV-17 E7 fused to a fragment containing the listerolysin O PEST domain. Their study showed that tumor regression was significantly more pronounced in the latter case. In 2009, this bacterial vector (*L. monocytogenes* carrying HPV-17 E7 fused to a fragment of listerolysin O) was evaluated in a Phase I Clinical Trial in patients with metastatic cervical cancer [78]. This constituted the first clinical trial using a live attenuated *L. monocytogenes;* the trial outcome demonstrated 30% tumor reduction and increased overall survival rate, and apart from minor flu-like symptoms and hypertension in some subjects the vaccine vector was well tolerated, indicating the safety and efficacy of listerial vectors.

Yang *et al.*, 2014 [185] reported a novel strategy involving genetic modification of replication-deficient *L. monocytogenes* to express and secrete the human CD24 protein. Overexpression of CD24, a glycosylphosphatidylinositol-anchored membrane protein, is correlated with poor therapeutic outcomes in some cancers, and contributes to experimental tumor growth and metastasis [186]. A further study also reported its role in promoting tumor cell invasiveness *in vivo*, and it serves as a hepatic cancer stem cell biomarker that has a strong association with apoptosis, metastasis and recurrence of hepatocelullar carcinoma [185,187,188]. In IV administered mice, the vaccine vector efficiently enhanced both Th1 and Th2 immune responses, indicated by enhanced production of IFN-γ, IL-4 and IL-10, resulting in significant reduction in tumor size and prolonged survival in mice. Of note was a reduction of T regulatory cell numbers, and enhanced specific CD8+ T-lymphocyte activity was observed in the tumor-infiltrating lymphocytes.

### 3.9. In Vivo Tumor Imaging

Despite the ongoing improvements in cancer therapy, early detection remains a vital aspect of effective treatment and better prognosis. Bacteria that preferentially replicate in tumors have been exploited in diagnostic applications, and genetically engineered bacterial vectors expressing imaging agents allow the detection of not only the primary tumor site but also metastatic sites [189,190]. As accumulation of bacteria in tumors occurs over time, the need to administer large quantities of probe for an enhanced signal/noise ratio can be eliminated [86]. Bacteria expressing light-emitting molecules could be visualized whilst colonizing tumors. Both fluorescent (Green Fluorescent Protein (GFP) and its variants) [191] and luminescent (*lux*) [192] genes are available for this strategy. Riedel *et al.*, 2007 [193] successfully developed a system for the stable expression of high level bioluminescence (luciferase) in *L. monocytogenes*, providing a platform for *in vivo* tumor-targeting studies. On the other hand, a study conducted by Xiong *et al.*, 2013 [191] using a GFP-carrying vector to transfect murine tumor models also showed promise. Other bacteria-based tumor-targeting imaging systems have also been examined, including Positron Emission Topography (PET) scanning in combination with bacteria expressing thymidine kinase [194]. Using this strategy, *E. coli* expressing endogenous thymidine kinase [195] and *S.* Typhimurium expressing thymidine kinase derived from HPV were evaluated [196,197]. Bacterial vector-mediated *in vivo* tumor imaging technology is a promising approach for the early detection of primary tumors as well as metastasis. Such technology also holds the key for real-time monitoring of disease progression and treatment efficacy, which would significantly enhance the prognosis and the quality of life of cancer patients.

## 4. Conclusions

Harnessing the ability of attenuated bacterial vaccines to elicit robust immune responses, the generation of recombinant vaccine vectors designed to protect against a heterologous pathogen is an attractive route for vaccine development. Some vectors are already in commercial use, and as we understand more about the pathogenicity and immune response induction by the vectors we can better tailor the vector to the antigen being expressed. This requires knowledge of the immune responses required to combat the pathogen from which the heterologous gene is derived, and marrying this to the delivery route and immune responses induced to the vector. However, bacterial vectors are not only useful for prophylactic vaccination. The use of these in combatting tumors is a very exciting and highly significant area of study, given the high burden cancers place on human health. As discussed, the intrinsic characteristics of different bacterial vectors give rise to particular strategies for anti-tumor therapeutics. This includes the ability of *Salmonella* to transfect tumor cells with therapeutic genes, the ability of *L. monocytogenes* to induce immune responses through the MHC Class I pathway, and the non-pathogenic nature of *Bifidobacterium* that can avoid potential side-effects. Each of these carries merit in the developing field of anti-tumor bacterial therapies. Overall, the development of live bacterial vectors for vaccination and anti-tumor therapies is a rapidly expanding research area with much potential. The future is bright for vectored vaccines. 
